# Non-pneumatic compression and its clinical utility in management of lymphedema. A position statement endorsed by the American Venous Forum and the American Venous and Lymphatic Society

**DOI:** 10.1016/j.jvsv.2025.102356

**Published:** 2025-11-19

**Authors:** Glenn R. Jacobowitz, Ruth Bush, Ronald S. Winokur, Joseph D. Raffetto

**Affiliations:** a Department of Surgery, Northwell Health, New York, NY; b Department of Surgery, UTMB Health, Galveston, TX; c Department of Radiology, Weill Cornell Health, New York, NY; d Department of Surgery, VA Boston Healthcare System, Boston, MA

**Keywords:** Lymphedema, Non-pneumatic compression, Phlebolymphedema

## Abstract

**Background:**

Lymphedema is a chronic, incurable condition caused by impaired lymphatic drainage, leading to progressive swelling and functional impairment. Phlebolymphedema, a subtype linked to chronic venous insufficiency (CVI), accounts for 41.8% of lower extremity lymphedema cases and contributes to an economic burden exceeding $1 billion over 5 years. Despite its high prevalence, lymphedema remains underdiagnosed and undertreated. Current management relies on compression therapy, exercise, and limb elevation, with pneumatic compression devices (PCDs) as adjuncts. However, PCDs present limitations, including user immobilization, poor adherence, and inadequate muscle pump activation, highlighting the need for more effective, patient-centered therapies.

**Methods:**

This paper evaluates the use of a non-pneumatic compression device (NPCD) that integrates static compression, sequential gradient compression (distal to proximal), and muscle pump activation.

**Results:**

In two randomized controlled trials, NPCD demonstrated superior outcomes over advanced PCDs, including greater edema reduction, improved quality of life, and higher adherence rates. Unlike traditional PCDs, NPCD enables active movement during treatment, optimizing lymphatic drainage and venous return.

**Conclusions:**

Given its distinctive multi-modal mechanisms and advantages, NPCD represents a clinically safe and effective, user-friendly alternative to PCDs, aligning with current best practices in vascular and lymphatic medicine. Its inclusion in future lymphedema treatment guidelines should be considered.

Lymphedema is caused by impaired lymphatic drainage, leading to fluid accumulation and swelling, primarily in the extremities. Prevalence estimates range from 5 to over 10 million^1^^,^^2^ affected individuals in the United States (U.S.). Health economic analyses estimate over $1 billion^3^ in lymphedema-related health care costs over 5 years.

Phlebolymphedema has emerged as a leading cause of lymphedema in the U.S., arising from chronic venous insufficiency (CVI), where elevated venous pressure disrupts lymphatic function. A study by Dean et al found phlebolymphedema in 41.8% of 440 lower extremity lymphedema cases, exceeding cancer-related cases (33.9%).^4^ The American Venous Forum (2022) recommends treating CVI (CEAP C3-C6) as lymphedema^5^ to improve management outcomes.

Despite its high prevalence and economic burden, lymphedema remains underdiagnosed and undertreated. Early intervention is critical for improving outcomes. Current guidelines emphasize conservative management, including limb elevation, exercise, and compression garments, with adjunctive use of pneumatic compression devices (PCDs). However, PCD limitations—complex application,^6^ patient immobilization, and poor adherence—contribute to disease progression, underscoring the need for improved treatment strategies.

This paper reviews the clinical evidence and real-world experience of a non-pneumatic compression device (NPCD), evaluating its role in addressing treatment challenges and improving lymphedema management.

## Non-pneumatic compression treatment

### Description

There is currently a U.S. Food and Drug Administration (FDA)-cleared, prescription-only NPCD indicated for the treatment of lymphedema, phlebolymphedema, venous insufficiency, lipedema, and chronic edema-related conditions. It supports active mobility during treatment, which can be administered in clinic or at home.^7^

The system consists of a compact, mobile-powered controller and limb-specific garments that deliver programmable, therapeutic sequential gradient compression (distal to proximal). Unlike traditional PCDs, the NPCD does not rely on stationary air compressors or air-inflated sleeves, offering a more streamlined, user-friendly alternative for edema management.

### Design and mechanisms of action

The NPCD differs from traditional PCDs in both design and mechanism of action. It features an inelastic compression garment embedded with electromechanical actuators with nitinol, a shape memory alloy. Upon activation by the controller, a small electrical current induces a phase transformation in the alloy’s crystalline structure, generating compressive pressure on the limb.^8^ The applied pressure (P) is directly proportional to the force (F) exerted over the contact area (A), as defined by: P = F/A. The NPCD’s controller ensures a consistent pressure gradient, mitigating fluid backflow concerns, while its wearable, electromechanical design provides a highly portable, noiseless, and responsive compression therapy.

The NPCD provides a clinically effective, multimodal alternative to PCDs, integrating evidence-based therapeutic modalities supported by clinical literature, guidelines,^9^ and consensus statements. NPCD treatment includes: (1) static compression via a lightweight, inelastic garment that conforms to the limb, ensuring consistent pressure and edema containment; (2) gradient sequential compression (distal to proximal) with adjustable therapeutic pressures (0-100 mmHg)^10^ to facilitate fluid drainage; and (3) support for mobility and exercise during treatment session, added benefits for enabling muscle pump activation to enhance lymphatic and venous return.

The wearable design allows active movement during treatment, promoting calf and thigh pump activation, which optimizes both lymphatic drainage and venous circulation. Microvascular fluid exchange is transient in most tissues, with filtration prevailing in the steady state in the venous system—making NPCD a more comprehensive approach to fluid management aligned with contemporary pathophysiological understanding.^11^

### Clinical advantages of NPCDs

Unlike PCDs, which require user immobilization in a supine position, NPCD enables users to continue activities of daily living during treatment. This distinction is important, as immobilization can impair optimal lymphatic and venous function, limiting lymphangion contractions essential for deep lymphatic drainage.^12^, ^13^, ^14^ The synergistic microcirculatory relationship between the lymphatic and venous systems underscores the importance of maintaining muscle activity and venous return, particularly in patients with phlebolymphedema.

NPCD has demonstrated superior clinical outcomes compared with advanced PCDs in two randomized controlled trials,^15^^,^^16^ achieving improved lymphatic fluid movement and enhanced muscle pump activation. For lower extremity fluid management, the calf muscle pump is responsible for most of the venous return to the central venous system, with a high capacitance and an ejection fraction of 65%.^17^ In venous reflux, retrograde flow increases venous volume up to five-fold, overwhelming the calf pump’s output and exacerbating symptoms. Exercise improves ejection fraction, enhancing venous return and reducing symptom burden in venous insufficiency.^17^^,^^18^ This effect is well-documented in both venous stasis and lymphedema, highlighting the interdependence of lymphatic and venous function and the role of fluid retention in fibrosis and pain.^19^^,^^20^

By addressing the limitations of PCDs, NPCD provides an evidence-based, patient-centric approach to lymphedema and phlebolymphedema management, aligning with current best practices in vascular and lymphatic medicine.

### Clinical evidence summary of significant trials

NPCD has been used to treat over 10,000 patients and has been clinically evaluated across 11 research studies.^15^^,^^16^^,^^21^, ^22^, ^23^, ^24^, ^25^, ^26^ These studies include qualitative imaging research, sizable open-label studies, and two large, prospective, multi-center, randomized, head-to-head comparative effectiveness trials,^15^^,^^16^ both of which demonstrated NPCD’s safety, efficacy, and clinical superiority over current of standard care, advanced pneumatic compression. Results from both trials have been published in the *Journal of Vascular Surgery, Venous and Lymphatic Disorders* (NILE^15^ and TEAYS^16^).

#### NILE^15^ (upper extremity)

A prospective, randomized, single crossover head-to-head evaluation was performed across five U.S. sites to compare the clinical outcomes for NCPD vs the standard of care, advanced pneumatic compression (APCD) in patients with upper extremity lymphedema. APCD involves the use of devices with programmable pumps and sequential inflation/deflation of multi-chamber garments. Patients with active cellulitis or skin ulceration were excluded from the study.

An initial 4-week washout period where no compression devices were allowed except for compression garments was instituted, after which patients were randomized to either NPCD or APCD treatment arm. Patients then used their assigned device for 28 days. This was followed by a second 4-week washout period, after which the patients were crossed-over to the treatment arm they were not initially assigned and proceeded to use the device for 28 days.

Primary endpoints included changes in mean reduction in limb edema volume and quality of life (LYMQOL) between baseline Day 0 and Day 28. Secondary endpoints include patient-reported adherence and safety. Additional data was on patient satisfaction was also collected.

At Day 28, patients using NPCD achieved greater mean reduction in limb edema volume vs using APCD (64.6% vs 27.7%; *P* < .05). On quality of life (overall LYMQOL score), patients reported a significantly greater mean improvement using NPCD (2.44; *P* < .01) compared with patients using APCD, in which there was no improvement. Patient reported adherence was reported as (95.6% NPCD vs 49.8% APCD; *P* < .01).

Patients reported greater satisfaction with NPCD (90% vs 14% APCD). In addition, 100% of patients indicated that use of the NPCD facilitated exercise compared with 0% with APCD. No device-related adverse events were reported. A subsequent subanalysis^23^ of patients limited to only those aged 65 or greater corroborated similar outcomes as those observed in the main analysis.^23^

#### TEAYS^16^ (lower extremity)

A prospective, randomized single crossover head-to-head investigation was performed across nine U.S. sites to compare the clinical outcomes for NCPD vs the standard of care, APCD, in patients with lower extremity lymphedema. Patients with active cellulitis or skin ulceration were excluded from the study.

An initial 4-week washout period where no compression devices were allowed except for compression garments was instituted, after which patients were randomized to either the NPCD or APCD treatment arm. Patients then used their assigned device for 90 days. This was followed by a second 4-week washout period, after which the patients were crossed-over to the treatment arm they were not initially assigned and proceeded to use the device for 90 days.

Primary endpoints included changes in mean reduction in limb edema volume and quality of life (LYMQOL) between baseline Day 0 and Day 90, as well as patient-reported adherence. Secondary endpoints included safety and patient treatment preference.

At Day 90, patients using NPCD achieved greater mean limb volume reduction vs using APCD (369.9 mL vs 83.1 mL; *P* < .05). On quality of life (overall LYMQOL score), patients reported experiencing greater mean improvement using NPCD vs using APCD (1.01 vs 0.17; *P* < .05). Patient-reported adherence was reported as (81% NPCD vs 56% APCD; *P* < .001). No device-related adverse events were observed.

Analysis of the subset of device-naïve (those who have never used a compression device) population of 31 patients in the NPCD treatment arm showed a mean limb volume decrease with standard error of 266.4 ± 92.02 mL (*P* < .05) was achieved vs that of 162.5 ± 76.14 mL (*P* < .05) for the APCD treatment arm. The overall LYMQOL score improvements of 1.15 ± 0.46 (*P* < .05) for NPCD vs that of 0.50 ± 0.34 (*P* > .05) for APCD were achieved. Treatment adherence was reported as 80.0% ± 4.3% for NPCD and 55.0% ± 6.7% for APCD, which follows the general trend observed in the greater patient population of the study.

Two additional subanalyses^16^ showed that, even when accounting for potential biases for the order of which device was first administered as well as for treatment adherence, those patients on NPCD achieved meaningfully better outcomes compared with when they were on APCD.

Trial health utilization data showed that patients using NPCD had no disease-related cellulitis, ulcerations, or hospitalizations, compared with 4% and 1% incidence of cellulitis and ulcerations, respectively, and an average of 8 hospital days for those on APCD.

### Real-world experience

Since its market introduction in 2021, NPCD has been generally well-received by practitioners and patients and is accessed through many regional and national commercial payers as well as Medicare, Medicaid, and Veterans Affairs. NPCD is described by active NPCD Healthcare Common Procedure Coding System (HCPCS) billing codes E0680 (controller) and associated codes E0678, E0679, and E0682 (limb accessories). These codes were established by the Centers for Medicare and Medicaid through the HCPCS coding process, which included evaluation of the FDA clearance and available clinical literature as well as public hearings. Through the issuance of these codes, Centers for Medicare and Medicaid recognized that NPCD mechanisms of action are therapeutically distinct from pneumatic compression and established a fee schedule comparable to that of an advanced pneumatic compression device (E0652). Potential drawbacks of NPCDs include localized skin irritation and compliance, and NPCDs may provide limited advantage in patients with impaired ambulation capability.

## Conclusions

Lymphedema remains a chronic condition that is often difficult to recognize and treat in the United States. Phlebolymphedema, lymphedema with venous etiology, has been shown to be a leading cause of this incurable condition. Recent passage of the Lymphedema Treatment Act brings new awareness to this condition as well as highlighting the need for treatments that address some of the clinical and practical treatment challenges associated with long-term management of this condition.

Non-pneumatic compression offers a therapeutically advanced hybrid approach by integrating the proven benefits of compression garments, sequential gradient compression, and muscle pump activation into a single treatment. This design optimizes fluid management, enhances adherence, and improves overall treatment effectiveness.

A 2020 systematic review of lymphedema interventions^9^ rated prescribed exercise (1C) and compression garments (1A) as treatments with a high degree of evidence, whereas intermittent pneumatic compression (PCD) was rated 2B. NPCD incorporates these evidence-based modalities into a single therapy, aligning with current pathophysiological understanding and guideline recommendations.

Published clinical evidence, including two randomized clinical trials, FDA clearance, and real-world patient data, supports NPCD as a safe, effective, and clinically meaningful treatment for lymphedema, phlebolymphedema, and related conditions when medically necessary. NPCD may deliver superior therapeutic outcomes compared with APCDs.

Given its multi-modal approach and patient-friendly design, NPCD should be considered after conservative treatment failure and included in future guideline assessments. By addressing common limitations of APCDs, NPCD provides an effective compression solution that enhances both lymphatic and venous return and offers clinicians access to a distinct, clinically validated, and patient-centric treatment option that improves outcomes while reducing barriers to adherence.

## Author contributions

Conception and design: GJ, RB, JR

Analysis and interpretation: GJ, RW, JR

Data collection: GJ

Writing the article: GJ

Critical revision of the article: GJ, RB, RW, JR

Final approval of the article: GJ, RB, RW, JR

Statistical analysis: Not applicable

Obtained funding: Not applicable

Overall responsibility: GJ

## Funding

None.

## Disclosures

None.
